# Practical Departments

**Published:** 1896-07-25

**Authors:** 


### PRACTICAL DEPARTMENTS.

### PEGAMOID.

Visitors to the Indian Exhibition are certain to have admired the "Pegamoid" rooms, fitted up in charming taste by Messrs. Wallace, of Curtain Road, though they may not have inquired into the special characteristics of the really wonderful invention which this pretty room is designed to show forth. Pegamoid is a preparation with which it is found possible so to treat wall-papers and hangings, cloth, leather, ticking, &c., as to render them absolutely washable, with the result that in the future re-papering and decorating will only be needed for the sake of variety. Cleanliness and disinfection are achieved by the simple application of soap and water, or such disinfectants as carbolic acid or solution of bichloride of mercury, which it has been satisfactorily demonstrated can be used without injury to the "pegamoided" substance. The process can be applied to linen, cotton, and woollen cloths, as well as to all papers; the material produced may be made of any desired colour, and afterwards can be polished and embossed as an imitation leather. There are numerous other uses. It may be employed for tents, carriage cloths, or belting, and renders cartridges effective in spite of weather.

It will be seen at once that for hospitals, nursing homes, and institutions generally, this invention will be extremely valuable. Pegamoid wall-papers, that can be thoroughly washed down with soap and water and strong disinfectants, and chair and sofa coverings to which the same vigorous methods can be applied without damage, will mean an avoidance of trouble and expense which will be almost incalculable.

And the initial cost will be hardly increased, for among the many advantages of Pegamoid its comparative cheapness is not one of the least. Wall-paper, for instance, can be had from 1s. 9d. a piece.

In appearance, too, there is absolutely no fault to find—as anyone examining Messrs. Wallace's exhibit at Earl's Court must confess. The Pegamoid material which is used for upholstery and for fancy articles, writing cases, photograph frames, &c., is indistinguishable from the best morocco leather, and for the walls the method can be applied to any paper chosen. There is, therefore, no limit in this direction to the uses of Pegamoid in future house decoration, and we especially commend the invention to the attention of hospital and institution authorities. A visit should be paid to the aforesaid room at "India," and all particulars can be obtained either there or from Messrs. Wallace, Curtain Road, E., the London agents for the Pegamoid Company.

